# Hermansky-Pudlak syndrome—rare type 10 with AP3D1 mutation

**DOI:** 10.1093/omcr/omaf184

**Published:** 2025-09-28

**Authors:** Vijayakumar Balaraddi, Ketaki Nawlakhe, Shilpa K, Prathik Bandiya

**Affiliations:** Department of Pediatrics and Neonatology, Indira Gandhi Institute of Child Health, Indira Gandhi Institute of Child Heath, South Hospital Complex, Banglore, Karnataka, 560029, India; Department of Pediatrics and Neonatology, Indira Gandhi Institute of Child Health, Indira Gandhi Institute of Child Heath, South Hospital Complex, Banglore, Karnataka, 560029, India; Department of Pediatrics and Neonatology, Indira Gandhi Institute of Child Health, Indira Gandhi Institute of Child Heath, South Hospital Complex, Banglore, Karnataka, 560029, India; Department of Pediatrics and Neonatology, Indira Gandhi Institute of Child Health, Indira Gandhi Institute of Child Heath, South Hospital Complex, Banglore, Karnataka, 560029, India

**Keywords:** oculocutaneous albinism, Hermansky-Pudlak syndrome, AP3D1 gene

## Abstract

Neonates with oculocutaneous albinism who exhibit additional systemic involvement need heightened clinical vigilance and prompt genetic testing. This is a case of a sick dysmorphic late preterm neonate with oculocutaneous albinism, hepatosplenomegaly, microcephaly, central hypotonia and severe encephalopathy, presenting since birth. Genetic analysis revealed AP3D1 gene mutation suggestive of Hermansky-Pudlak Syndrome (HPS) type 10. Severe neurological involvement in HPS is highly suggestive of type 10, indicating poor outcome. This case report aims to give a comprehensive account of the patient’s clinical course and offer prognostic insights and guidance that may be applicable to such analogous neonatal cases.

## Introduction

Hermansky-Pudlak syndrome (HPS) is a multisystemic genetic rare disorder manifesting primarily with oculocutaneous albinism, predisposition to bleeding due to platelet dysfunction, and in some cases, additional systemic manifestations such as restrictive lung disease, granulomatous colitis, and neutropenia. Genetic diagnosis and sub-typing are crucial for foreseeing the complications, management, and prognostication. This case describes a neonate with AP3D1 gene mutation, recently classified as HPS type 10, which presented at birth with microcephaly, encephalopathy, multiple joint contractures, hepatosplenomegaly, which emphasizes the necessity for heightened clinical vigilance and prompt genetic testing.

## Clinical description

We present a case of a girl baby born at 36 weeks 5 days gestation with 2800 g weight at birth. Mother’s first born is a well child; the second succumbed on day 1. Cause of death was attributed to birth asphyxia and syndromic features, and not evaluated further. During this pregnancy, the mother had oligohydroamnios, a normal anomaly scan, and breech lie, for which elective LSCS was done.

**Figure 1:**
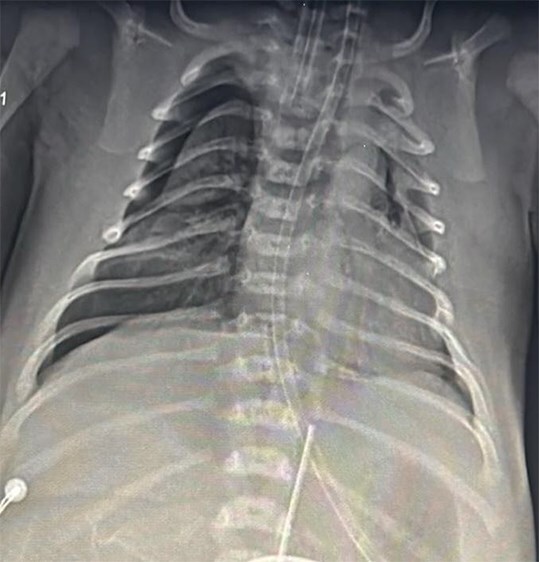
X ray chest showing pneumothorax.

The baby did not cry after birth, so bag and mask ventilation was done for 60 seconds, and the baby was subsequently intubated for poor respiratory efforts. The baby was transferred to a higher-level center on the same day. The baby had a high oxygen requirement in spite of two doses of surfactant. Also developed pneumothorax on day 2, resolved after intercostal tube (ICD) placement (Fig. 1).

The baby did not experience any seizures during the first week of life. Clinical seizures began on day 7 and required treatment with three antiepileptic drugs (AEDs).

**Figure 2:**
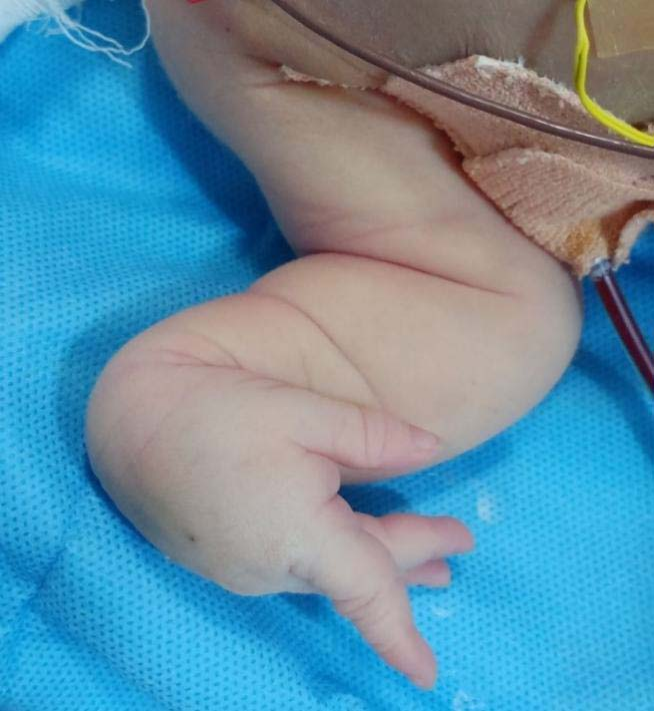
Congenital contracturs of the wrist joint.

**Figure 3:**
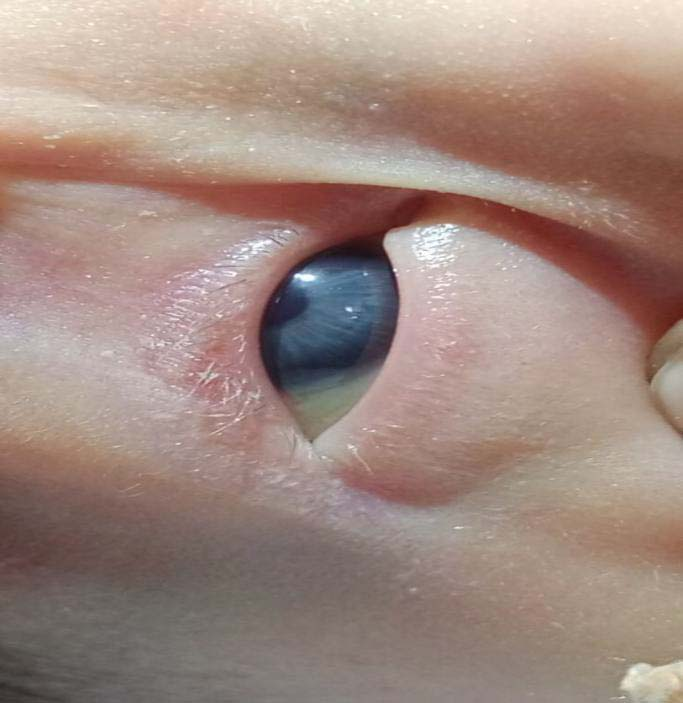
Hypo-pigmented iris.

**Figure 4:**
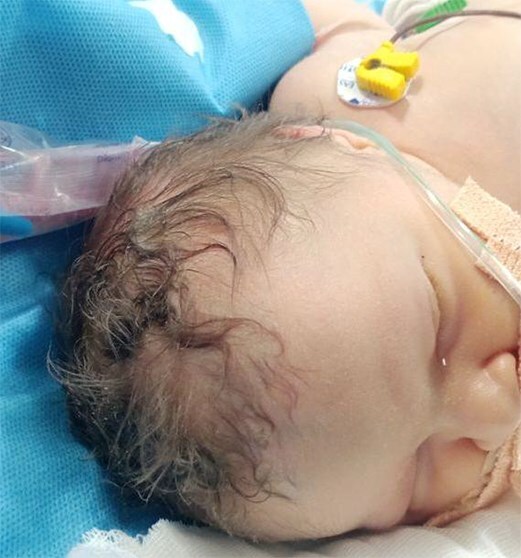
Hypo-pigmented hair.

The baby had severe encephalopathy since birth, generalized hypotonia and microcephaly. Baby had syndromic features—oculocutaneous albinism, hypo-pigmented hair, hypo-pigmented iris, microcephaly with head circumference 30 cm, very small anterior fontanelle admitting just the tip of a finger, no eyebrows, micrognathia, low-set ears, flat nasal bridge, contractures at both wrist joints, left club foot. (Fig. 2–4) The baby had central hypotonia and developed hepatosplenomegaly around the second to third week of life.

Investigation—An Altered coagulation profile was noted on day one, which was corrected after a fresh frozen plasma transfusion. Platelet abnormality or further coagulopathy was not noted. The initial neurosonogram(NSG) was normal on day 2. Repeated NSG on day 10 of life showed cerebral edema and bilateral echogenic basal ganglia.

We were unable to conduct the hearing evaluation and NK cell activity due to financial constraints.

Whole-exome sequencing confirmed AP3D1 gene splice donor variant of c.1859 + 1G > T with Genomic Nomenclature of chromosome 19:g.2117220C > A, which is consistent with HPS type 10.

By the 20th day of life, the baby’s condition continued to deteriorate. The infant continued to exhibit severe encephalopathy and required high support from invasive mechanical ventilation. Parents decided to take the baby to the nearby hospital, where the baby expired in 48 hours.

## Discussion

Hermansky-Pudlak syndrome inherits in an autosomal recessive manner with a global prevalence of 1 in 500 000 to 1 000 000 people [1]. Commonly manifests with albinism, platelet and immune dysfunction, and affects other systemic organs due to lysosomal accumulation of ceroid lipofuscin. Genetic mutation alters the formation of lysosomes and lysosome-associated organelles, resulting in impaired protein transport [2].

HPS is a platelet storage pool deficiency disorder. Usually, platelets have alpha and delta or ‘dense’ granules. Latter is a lysosome-related organelle and contains specific key molecules that are discharged immediately for the second aggregation response. This process is disrupted in HPS, leading to bleeding. Other mimics of HPS are Chediak-Higashi syndrome, Wiskott-Aldrich syndrome, and Griscelli syndrome. In the presence of typical clinical features, the absence of delta granules in the platelets on an electron microscope confirms the diagnosis. This bleeding tendency can present with a spectrum of severity ranging from bruising to prolonged bleeding. Generally, not seen in the immediate neonatal period.

Impaired melanosome biogenesis and transport to keratinocytes results in hypopigmentation. The skin and hair colour are variable. Older individuals do have solar skin damage along with a high risk of skin cancer. The ocular findings include iris and retinal hypopigmentation, early-onset nystagmus, and photophobia [3].

Immunodeficiency is often seen in patients with HPS, especially in types 2 and 10. NK cells have reduced cytolytic activity, and neutrophils exhibit impaired intracellular elastase content and increased expression of CD63 on the plasma membrane. These defects contribute to the immunodeficiency. Additionally, there is dysfunction of cytotoxic T cells and dendritic cells.

Ceroid lipofuscin is an abnormal fat containing a lysosomal pigment complex, which can damage cells and lead to various organ complications, including pulmonary fibrosis and granulomatous colitis.

There are eleven different types of Hermansky-Pudlak syndrome, which can be distinguished by their signs and symptoms, and the underlying genetic cause. Mutations in BLOC-1 (biogenesis of lysosome-related organelles complex 1) manifest with HPS types 7, 8, 9, and 11. BLOC-2 gene mutation leads to HPS subtypes 3, 5, and 6, which are milder variants. BLOC-3 gene mutations are seen in HPS 1 and 4 subtypes, and AP-3 (Adaptor Protein complex 3) gene mutations are seen in HPS 2 and 10, manifesting with profound multiorgan involvement [3].

The baby had mutations in the AP3D1 gene, which expresses the delta subunit of the AP-3 complex. AP-3 complex is a heterotetramer, has four different subdivisions: smaller sigma (σ), medium (μ), and two large (delta, δ, and beta β3A). Adaptor protein complex primarily facilitates the sorting and trafficking of proteins to lysosomes and related organelles, like melanosomes and platelet-dense granules.

HPS-2, caused by mutations in AP3B1A, leads to a highly similar clinical presentation to that of type 10, but lacks CNS involvement. Delta subunit mutation has severe neurological involvement, including intractable seizures and microcephaly.

Ammann et al. in 2016 first reported this novel mutation in a male infant, highlighting its phenotypic similarities to the ‘mocha’ mouse model, which is associated with a null mutation in the AP3D1 gene [4]. A similar mutation in the AP3B1A gene has been identified in three affected individuals from the same consanguineous family [5]. To our knowledge, to date, only four cases of Hermansky-Pudlak Syndrome (HPS) type 10 have been reported, and we are presenting the fifth case.

Genetic counseling is recommended for families with a history of HPS to understand the risks and inheritance patterns.

While there is no permanent restoration for HPS, supportive treatments in a few subtypes can help to improve quality of life. Notably, HPS type 10 with severe neurological involvement can significantly impact life expectancy, with many complications leading to death.

## Conclusion

This case highlights the crucial role of genetic tests and sub-typing the diagnosis of dysmorphic albinism babies for prognostication. HPS with multisystemic involvement, especially CNS, has high mortality in the neonatal period.
